# Sperm storage causes sperm senescence in human and non-human animals

**DOI:** 10.1098/rspb.2025.3181

**Published:** 2026-03-25

**Authors:** Krish Sanghvi, Rebecca Dean, Shinichi Nakagawa, Klaus Reinhardt, Irem Sepil, Regina Vega-Trejo

**Affiliations:** 1 Department of Biology, University of Oxford, Oxford OX1 3EL, UK; 2 University of Alberta, Edmonton, Alberta T6G 2E9, Canada; 3 Biology, Applied Zoology, Technische Universität Dresden, Dresden 01069, Germany

**Keywords:** IVF, reproductive biology, sexual abstinence, sexual selection, sexual conflict, sperm selection, ejaculate, inter-generational effects, Evolution, evolution, physiology, ecology

## Abstract

In animals, mature sperm are stored in males before ejaculation and sometimes in females before fertilization. Sperm storage provides evolutionary advantages; however, storage can also cause sperm deterioration owing to post-meiotic sperm senescence (PMSS). Yet, the extent of such deterioration, the mechanisms driving it and its fitness consequences remain poorly understood. We perform a meta-analysis across human (115 studies) and non-human animals (56 studies, 30 species) to understand the impacts of *in vivo* sperm storage. In men, we find that storage via sexual abstinence increases sperm oxidative stress and DNA damage, while reducing sperm viability and motility. In non-human animals, sperm storage in males or females reduces sperm performance, including fertilization success and embryo quality. In men, the method used for sampling individuals—and in other animals, the sex-specific duration of storage—modulates observed outcomes. Our results highlight the mechanisms of sperm deterioration during storage and its consequences for pre- and post-fertilization outcomes. These have important implications for fertility clinics, sperm selection, captive breeding and understanding evolved adaptations that mitigate stored-sperm deterioration. Importantly, our results can help to optimize the timing of ejaculate collection and storage.

## 1 Introduction

Across the animal kingdom, males store mature spermatozoa for extended durations between spermatogenesis and ejaculation, while internally fertilizing females do so between mating and fertilization [1–3]. For instance, males of some bat [4,5], salamander and frog [6] species store mature sperm over many months of hibernation, while queen Hymenoptera and females of some reptiles store sperm for many years [1,7–9]. In males, sexual rest separates sperm production from sperm delivery, allowing sperm to accumulate, thus reducing the risk of sperm limitation during mating [10–12]. In females, sperm storage reduces costly re-mating, supports oviposition site selection, facilitates sperm competition and cryptic female choice and prevents sperm limitation when males are scarce [1,12–15]. However, storage in males or females can lead to temporal deterioration in the function of individual sperm (post-meiotic sperm senescence (PMSS), potentially impacting fertilization success and post-fertilization outcomes [2,3,16,17].

There are several physiological mechanisms that explain why sperm senesce during storage. Unlike other cell types, mature sperm stored *in vivo* are transcriptionally silent, possess minimal cytoplasm, are often highly motile and have poor access to ATP [18]. The high metabolic activity of sperm without ready access to glycogen and lipids can rapidly lower sperm viability and motility owing to the consequent depletion of energy reserves [18]. For instance, sexually rested guppy males have less motile and slower sperm and lower fertilization success than recently mated males [19–21]. Similarly, queen ants lose viability of sperm stored over many years [7]. The lack of cytoplasm and low transcription also severely limit the intrinsic capacity for sperm to repair DNA [16,22–25] or to produce endogenous antioxidants, making sperm highly vulnerable to damage from reactive oxygen species (ROS) [26,27]. Such intracellular damage of sperm DNA, mitochondria and membrane proteins and lipids owing to ROS can potentially impact embryos and offspring [17,22]. For instance, maternal [27,28] and paternal [29] sperm storage in some birds reduces embryo survival, while paternal sperm storage in fish and fruit flies lowers the reproductive success of sons [10,20]. Despite several studies finding evidence for PMSS, some have not found evidence for sperm storage impacting sperm performance positively or negatively [30–32], making the overall, animal-wide impacts of storage ambiguous.

One biologically important modulator of sperm deterioration during storage is the sex of the sperm-storing individual. Both males and females in several species have specialized sites or organs where sperm are stored or accumulated. In male mammals such examples are the epididymis, in male birds, the vas deferens and in male insects, the seminal vesicles [10,33]; while female arthropods use the spermathecae, female birds, the sperm storage tubule and female reptiles, the oviduct [1]. Generally, in both males and females, these sites/organs have similar functions [33]. They delay sperm activation, regulate pH, secrete antioxidants, reduce hypoxia, delay capacitation, limit oxidative phosphorylation, suppress immune surveillance, counteract ROS and stabilize sperm membranes [1,13,33]. However, sperm storage in males and females also differs in several ways. In males, an individual sperm is generally stored for shorter durations—several days in vertebrates to several weeks in arthropods, bats and amphibians [2–6,12,33]—before being reabsorbed [34] or ejaculated. Males of some species even spontaneously discard old sperm through masturbation and sperm dumping [35–38], therefore preventing the accumulation of old sperm. Furthermore, some males produce seminal fluid with proteins and antioxidants that can extend the longevity of stored sperm when both are mixed during storage [39–41]. In males, continuous spermatogenesis can further prevent increases in the average ejaculate age, even though individual stored sperm are ageing during storage [3]. Female storage, on the other hand, has evolved to facilitate much longer storage durations in some species. For example, female non-Chiropteran mammals store sperm for several days, female birds and many insects for several weeks, female bats and reptiles for many months and hymenopteran queens for many years [1,33]. Females have therefore evolved more specialized organs to extend sperm viability and facilitate longer-term storage [1,42–46]. Cells from these organs/sites often secrete reproductive fluid containing antioxidants and sources of energy [1,33,42–48]. Females can also employ behavioural strategies to reduce PMSS in stored sperm, for instance, by biasing the storage of sperm and fertilization of eggs towards younger sperm [43,44]. Females can also increase their rate of re-mating for fresh sperm when stored sperm accumulate high levels of oxidative stress [49,50]. Thus, while both sexes have several common behavioural and physiological strategies, they also differ in many ways, making sex a potentially important modulator of PMSS.

The type of outcome measured is also likely to be an important modulator, with some sperm traits being more susceptible to damage than others [51,52]. For example, sperm storage might compromise sperm performance and fertilization success more than post-fertilization outcomes. This could occur if post-fertilization mechanisms repair DNA damage in fertilized zygotes, or if damaged sperm are selectively completely excluded from fertilization (reviewed in [52]). Methodological factors, such as differences in the sampling method used to assay individuals, might also modulate the effects of sperm storage. Here, longitudinal sampling of the same individual across different sperm storage durations would minimize the impact of genetic and environmental differences, potentially leading to a higher detectability of PMSS compared with cross-sectional sampling [52].

Assessing the overall extent of sperm senescence during sperm storage requires synthesizing data across taxa and both sexes, while combining theory from biomedical and evolutionary fields and considering various moderators to explain observed patterns. Here, we conduct such a meta-analysis across humans and non-human animals to address three aims: (i) to document the effects of *in vivo* sperm storage on sperm and reproductive outcomes; (ii) to assess the impact of different moderating variables—specifically, the sex of the sperm-storing individual, type of sampling and sperm storage duration; and (iii) to compare the impacts of sperm storage on sperm performance versus post-fertilization outcomes.

## 2 Material and methods

We conducted and reported our meta-analysis following the PRISMA-EcoEvo guidelines [53] and performed all statistical analyses using R v. 4.1.2 [54]. We searched, screened and analysed humans versus non-human animal studies separately, given the stark biological and methodological differences between the two types of studies, and because there were substantially more studies found on humans.

### (a) Search protocol

We generated appropriate search strings to find relevant papers separately for human and animal studies, based on commonly used words found in the abstracts and keywords of previous studies on this topic (electronic supplementary material text S1, electronic supplementary material, figure S1 for search string). To obtain human studies, we used SCOPUS and the Web of Science on 26 January 2024. We also performed backward and forward searches for papers using six systematic reviews on male sexual abstinence (electronic supplementary material text S2) and identified two further studies from other sources. For obtaining studies on non-human animals, we used SCOPUS and Web of Science on 21 September 2021 and 12 October 2022, respectively, and a backward search using 11 relevant reviews (electronic supplementary material text S2). For non-human animals, we additionally included eight studies identified through other sources (electronic supplementary material, figure S1). After removing duplicates, we obtained 1069 human and 7559 animal studies. The abstracts of these were screened to assess their relevance for our meta-analysis.

### (b) Screening criteria

Abstracts for both human and non-human animal studies were evaluated using predefined inclusion and exclusion criteria, using the software Rayyan [55]. Studies were excluded if they: addressed irrelevant topics, only reported the presence or absence of sperm storage, focused solely on the descriptive biology of spermatogenesis or sperm storage organs, were case studies with a sample size of one, involved plants (e.g. on endosperm, meiosis in sperm), had overlapping sperm storage durations between groups, were not written in English, compared sperm storage across species rather than within-species and were reviews or meta-analyses. This initial screening identified 315 relevant studies on humans and 177 on non-human animals, which were subsequently subjected to full-text screening.

During the full-text screening conducted by two analysts, we added several exclusion criteria in addition to the criteria detailed above. Here, we further excluded studies that: stored/aged sperm *in vitro* or *ex*-*vivo* (i.e. outside the live individuals via cryopreservation or in a medium, e.g. [56]); addressed unrelated topics (e.g. sperm precedence, alcohol abstinence); were inaccessible; did not include variation in, or manipulation of, sperm storage durations; had unclear experimental designs; measured traits without a clear relation to sperm performance or post-fertilization outcomes (e.g. offspring sex ratio); and lacked sufficient data for calculating effect sizes (e.g. missing sample sizes). Importantly, we also excluded studies where outcomes could be potentially attributed to variation in sperm quantity or to ejaculate depletion. This is because our focus was on PMSS, which is a consequence of temporal changes in the quality of individual sperm during storage [2,3]. Thus, unless sperm numbers available during fertilization were controlled for across sperm storage treatments, traits like competitive fertilization success, sperm precedence or the number or proportion of eggs fertilized were excluded (e.g. [57]). Sample sizes of excluded studies and associated reasons are provided in electronic supplementary material text S1.

### (c) Data collection

To investigate the effects of *in vivo* sperm storage, we collected data on the means, standard deviations (s.d.) or standard errors (s.e.) and sample sizes of individuals for each sperm storage group/treatment (electronic supplementary material text S3). We additionally recorded the number of unique and total individuals sampled per study. The total number of individuals sampled exceeded the number of unique individuals in longitudinal (i.e. individuals sampled repeatedly at different durations of sperm storage) and semi-longitudinal studies (some individuals were repeatedly sampled). In human studies, all data focused on male sperm storage owing to a lack of studies on female storage, and our sample sizes were based on the numbers of males sampled. In non-human animal studies, the sample size corresponded to the numbers of individuals of the sex that stored sperm. When means and s.d./s.e. were not reported, we extracted test statistics (e.g. *t, F, χ*^2^ -values or *R*^2^) to compute effect sizes, following [58–60]. To ensure the accuracy of the data collection procedure and prevent analyst bias, two analysts independently screened the full texts and collected the data. Repeatability between them was high (electronic supplementary material text S4).

To examine the influence of moderators, we collected data on various biological and methodological variables from included studies. For human studies, these were: country, trait (electronic supplementary material text S5), health condition of males (fertile, infertile-attended infertility clinics, or undergoing treatment for other conditions), sperm storage duration range (i.e. difference between the maximum and minimum storage duration for each effect size, directly from the study) and sampling method (longitudinal, semi-longitudinal or cross-sectional). For non-human animal studies, our moderators included: species; taxonomic class; trait (electronic supplementary material text S5); sperm storage duration range; sex of the sperm-storing individual; sampling method and population type (laboratory, captive, wild or domestic). Furthermore, we collected data on the maximum lifespans of included animal species (electronic supplementary material text S6). Data on sperm storage durations for both humans and non-human animals were log_e_ transformed for normality (electronic supplementary material text S6, electronic supplementary material, figures S2 and S3).

Overall, our meta-analysis contained data on how sperm storage impacts three broad outcomes (electronic supplementary material text S5): (i) sperm intra-cellular quality (i.e. oxidative stress and DNA quality), which represented the mechanisms of sperm damage; (ii) sperm performance (i.e. motility, velocity, viability, morphology, fertilization success), which represented within-generation outcomes of sperm storage; (iii) post-fertilization outcomes (i.e. embryo or offspring quality), which represented the *inter-generational outcomes* of sperm storage.

### (d) Effect size calculation

We first calculated Pearson’s correlation coefficients using four different methods: (i) standardized mean differences for comparisons of two storage groups with reported s.d./s.e. (electronic supplementary material text S7A); (ii) simulations for multiple storage duration group comparisons with reported means, s.d./s.e. (electronic supplementary material text S7B); (iii) test statistics (e.g. *t*-values, *Z*-values) (electronic supplementary material text S7C); (iv) percent/proportion data when s.d. or s.e. was unavailable (electronic supplementary material text S7D). We then calculated Fisher’s *z*-transformed correlation coefficient (*Zr*) as our effect size from the calculated Pearson’s *r* [58] (electronic supplementary material text S7E). Effect sizes were standardized using a multiplier so that negative values indicated negative outcomes with increasing sperm storage, while positive values indicated positive outcomes. Sampling variance of effect sizes was calculated as: *VZ*_*r*_ = 1/(*N*_*e*_ – 3), where *N*_*e*_ was the effective sample size (electronic supplementary material text S8). For longitudinal and semi-longitudinal studies, *N*_*e*_ accounted for pseudo-replication of individuals sampled repeatedly [61].

### (e) Data analysis

We analysed human and non-human animal datasets separately. For both, we first constructed a meta-analytical model to assess the overall effect of sperm storage using the *rma.mv* function in the *metafor* package [62]. In all models, *Zr* was the response variable. We computed a variance–covariance (VCV) matrix with the *vcalc* function to estimate effect size variances, containing *VZr* (diagonal) and within-cohort covariances (off-diagonal, rho = 0.8) and used this matrix as the sampling variance [63,64]. Random effects included in both models for the VCV matrix were: effect size ID (residual variance), cohort ID (within-study variance) and study ID (between-study variance). For human data, we also included country of origin of the sampled men as a random effect to address possible non-independence caused by geography and culture. For animal data, we instead included species ID as well as a correlation matrix of phylogenetic relatedness as random effects, to control for relatedness and repeated measures within each species. The phylogenetic tree was constructed with the *ape* [65] and *rotl* [66] packages, and branch lengths were estimated using Grafen’s method [67]. The *i2_ml* function in the *orchard* package [63] was used to calculate whether the heterogeneity was significent (*I*^2^).

Next, we constructed meta-regressions to explore how moderators influenced the effects of sperm storage. ‘Full models’ for each dataset included all moderators relevant to that dataset. For the human dataset, these were: trait, health condition, sexual abstinence duration (absolute) and sampling method; for the non-human animal dataset, these were: trait, sex of storage, taxonomic class, male sampling method, sperm storage duration and population type. When analysing the effect of sperm storage duration in non-human animals, one analysis used the absolute values of sperm storage duration taken directly from the study, while a second analysis used the sperm storage duration relativized to the species’ maximum lifespan (electronic supplementary material, text S6). We used full models to assess the proportion of heterogeneity explained by all moderators while controlling for collinearity and confounding [68] (electronic supplementary material, figure S4). We additionally created individual meta-regressions for each moderator separately to test their independent effects. Here, models without intercepts quantified whether each level of a moderator was positively or negatively impacted by storage. Models with intercepts tested whether moderators explained significant variance and whether storage impacted two levels of the moderator differently. For all meta-regressions, we calculated total heterogeneity (*Q*_*E*_), heterogeneity explained by moderators (*Q*_*M*_), and marginal *R*^2^ for moderators (significance of α = 0.05). Effect sizes in §3 are presented as Pearson’s *r* (back-transformed from *Zr*) for ease of interpretation.

### (f) Publication bias

We assessed publication bias separately for human and non-human animal datasets using the following methods [69,70]: (i) funnel plots, to visually assess whether less precise studies with non-significant results were missing; (ii) trim-and-fill analysis, with study-level aggregation of effect sizes to quantify missing studies; (iii) time lag bias analysis, to test whether year of publication influenced *Zr*; (iv) small-study bias analysis, to test whether the sample size of a study influenced *Zr*; (v) selection model, to test whether studies with larger *Zr* were more likely to be included.

## 3 Results

We obtained 453 effect sizes from 115 studies, representing at least 54 889 unique men from 31 countries, on how sperm storage in men impacts sperm performance, sperm intra-cellular quality and post-fertilization outcomes. We also obtained 235 effect sizes from 56 studies representing 30 species for how sperm storage in male or female non-human animals impacts reproductive outcomes.

### (a) Humans

**Figure 1:**
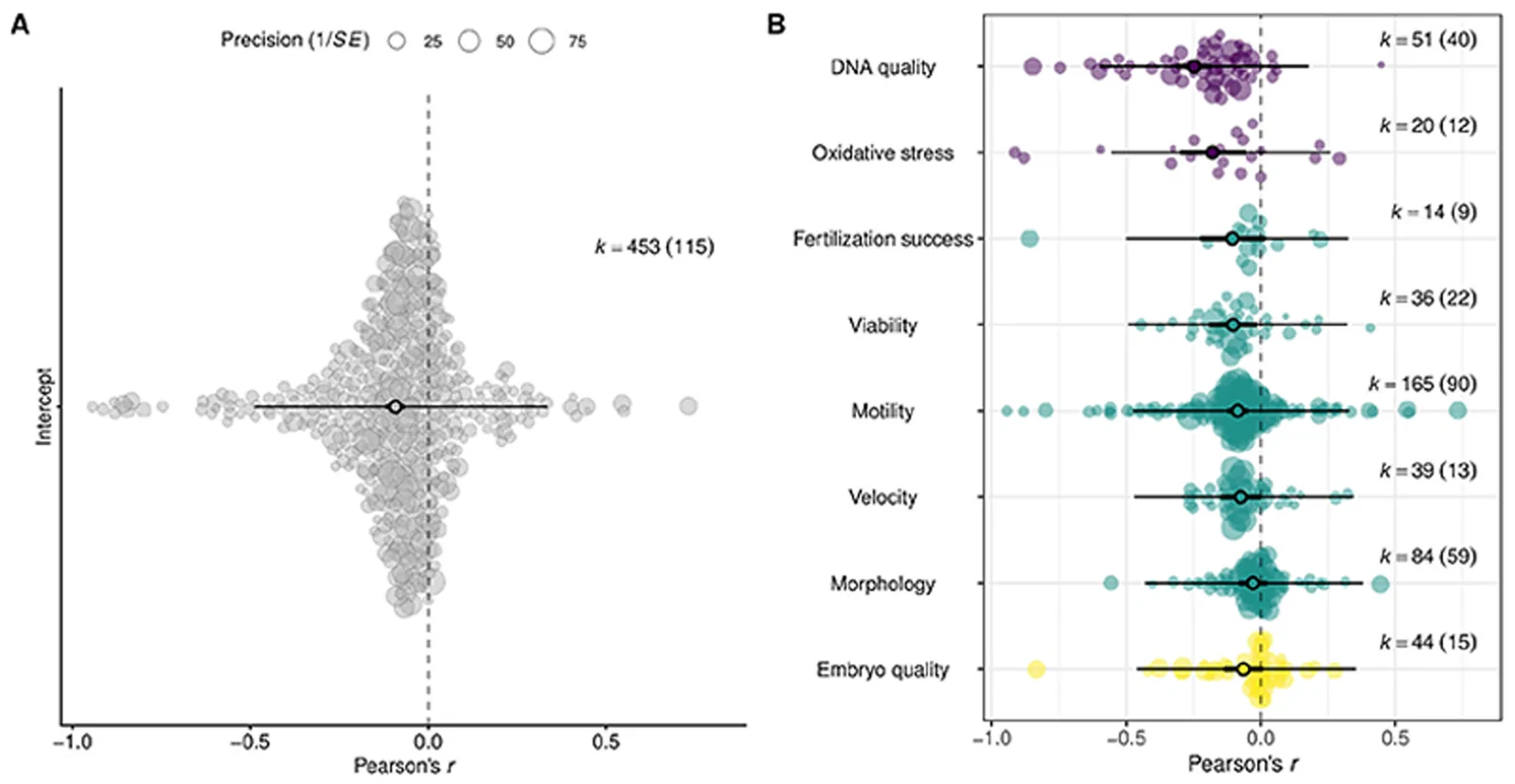
Overall and trait-specific effect of sperm storage in men. (A) Null model shows overall deleterious effects of sperm storage. (B) Consequences of sperm storage for different sperm intra-cellular quality (purple), sperm performance-related (green) and post-fertilization (yellow) outcomes. Negative Pearson’s *r* shows deleterious effects and positive Pearson’s *r* shows improvements in reproductive outcomes with storage. Size of each datapoint represents precision of the effect size. Bold error bars show confidence intervals (CI); light bars show prediction intervals (PI). Sample sizes *k* = number of effect sizes (number of studies in brackets). *SE* denotes standard error.

Sperm storage had a weak, but significantly negative impact on overall outcomes in men (Pearson’s *r* = −0.093 (CI: −0.120 to −0.066), figure 1A). There was significant heterogeneity in our data (*I*^2^ = 97.593, *p* < 0.001), most of which was attributed to differences between effect sizes (i.e. residual variance), with 0% of heterogeneity being explained by true differences between studies (electronic supplementary material, figure S5), country or cohort. All moderators collectively explained a significant proportion of variation in outcomes (*R*^2^ = 12.693%; *Q*_*M*_ = 40.691, *Q*_*E*_ = 30060.027, *p* < 0.001, *DF*_11 431_).

Trait-specific impacts of sperm storage explained significant heterogeneity (*Q*_*M*_ = 30.182, *Q*_*E*_ = 30524.926, *DF*_7,445_, *R*^2^ = 7.956%, *p* < 0.001). Sperm motility and viability significantly decreased, while sperm DNA damage and oxidative stress significantly worsened during storage (figure 1B). Embryo quality, fertilization rate, sperm velocity and morphology were not significantly impacted (figure 1B). Importantly, studies that sampled the same men longitudinally found more severe effects of sperm storage than cross-sectional studies where different cohorts of men represented different storage durations (electronic supplementary material, figure S6A; *Q*_*M*_ = 12.389, *Q*_*E*_ = 34544.397, *DF*_1,451_, *R*^2^ = 5.649%, *p* < 0.001). Neither the specific range of time for which men were sexually abstinent across studies (*Q*_*M*_ = 0.007, *R*^2^ = 0; *p* = 0.934, electronic supplementary material, figure S6B) nor the males’ health condition (*R*^2^ = 0%, *p* = 0.897, electronic supplementary material, figure S7) explained significant heterogeneity.

### (b) Non-human animals

**Figure 2:**
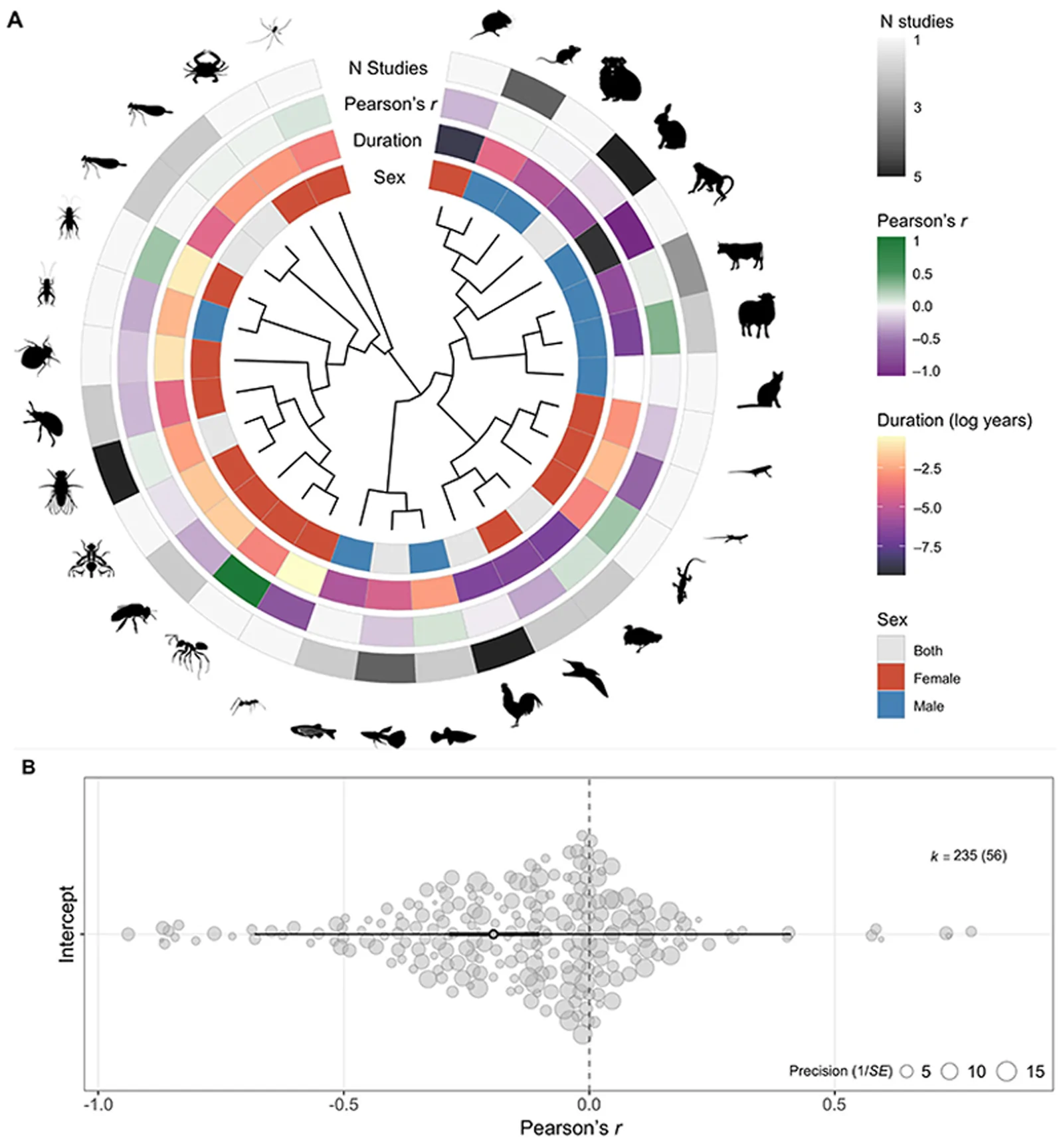
Overall and species-specific effects of sperm storage in other animals. (A) Phylogenetic tree of species included in the non-human animal dataset, specifying the sex of the sperm-storing individual, the duration of sperm storage (in loge years), mean effect size for each species and the number of studies. 1 hour ≈ −9.079 loge years, 1 day ≈ −5.9 loge years, 1 week ≈ −3.96 loge years, 1 month ≈ −2.485 loge years, 1 year ≈ 0 loge years. Branch lengths were calculated using Grafen’s method [67]. (B) Null model shows overall negative effects of sperm storage in non-human animals. Negative Pearson’s *r* shows deleterious effects, and positive Pearson’s *r* shows improvements in reproductive outcomes with storage. Bold error bars show CI, light bars show PI. Size of each datapoint represents precision. Silhouettes taken from Phylopics under CC0, CC-BY-3.0, CC-BY-4.0, CC-BY-NC-SA-3.0, CC1.0 licences.

Studies on non-human animals also found high heterogeneity (*I*^2^ = 82.782%, *p* < 0.001), with 43.104% attributed to true differences between studies (electronic supplementary material, figure S8), 4.513% to between-species differences, but 0% to between-cohort differences or phylogenetic relatedness (figure 2A). Here too, sperm storage led to significant, moderate overall deterioration (Pearson’s *r*: −0.198 (CI: −0.293 to −0.102), *z* = −4.057, *p* < 0.001, figure 2B). All moderators collectively explained substantial heterogeneity in outcomes (*R*^2^ = 22.274%), although this was marginally non-significant, probably owing to low statistical power and the large number of moderators (*Q*_*M*_ = 22.775, *Q*_*E*_ = 1194.243, *p* = 0.089, *DF*_15 214_).

**Figure 3:**
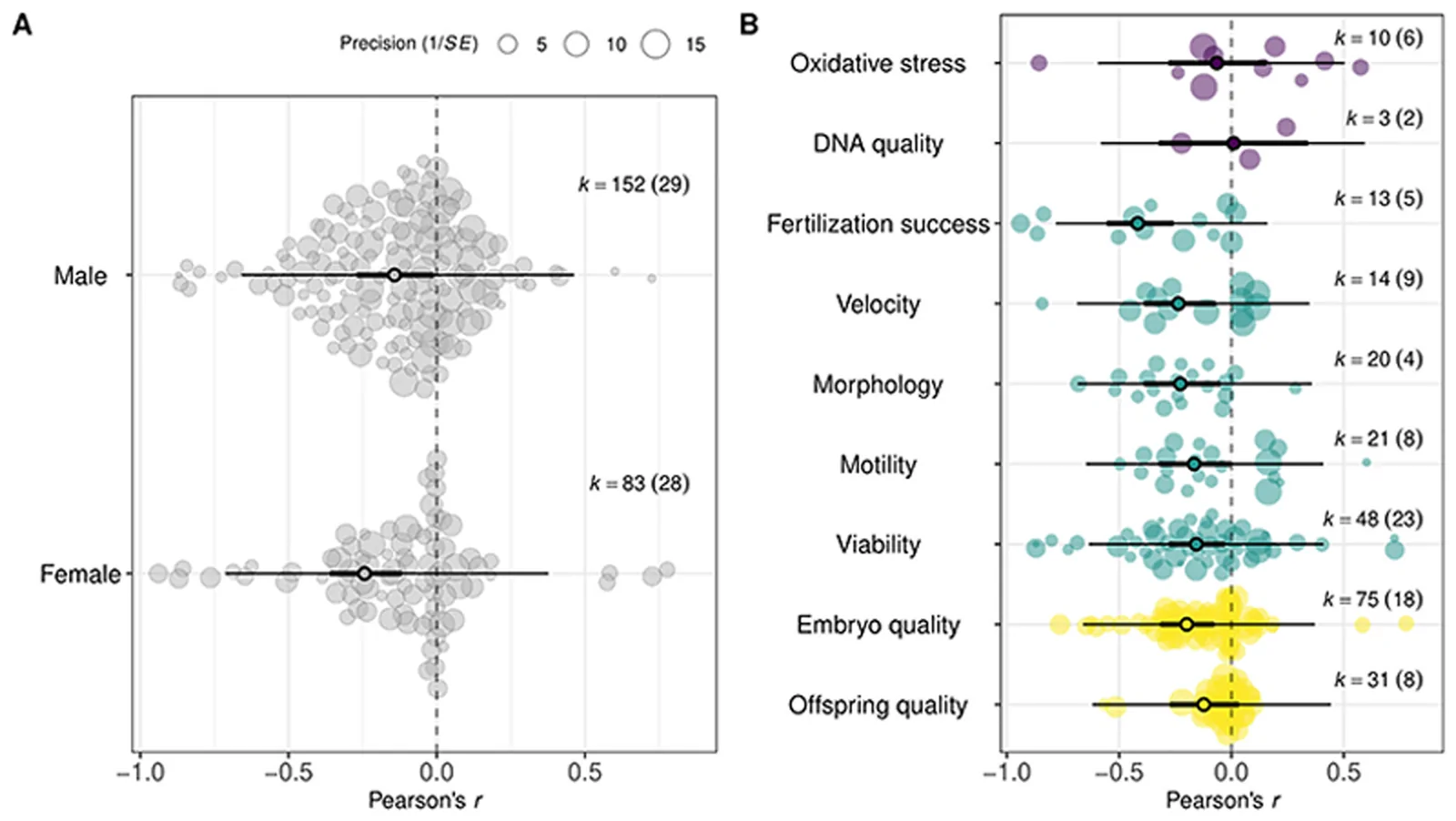
Sex- and trait-specific effects of sperm storage in non-human animals. (A) Overall deleterious effects of sperm storage in male and female animals. (B) Consequences of sperm storage for sperm intra-cellular quality (purple), sperm performance-related (green) and post-fertilization (yellow) outcomes. Negative Pearson’s *r* shows deleterious effects, and positive Pearson’s *r* shows improvements in reproductive outcomes with storage. The size of each datapoint represents precision. *k* = number of effect sizes (number of studies).

**Figure 4:**
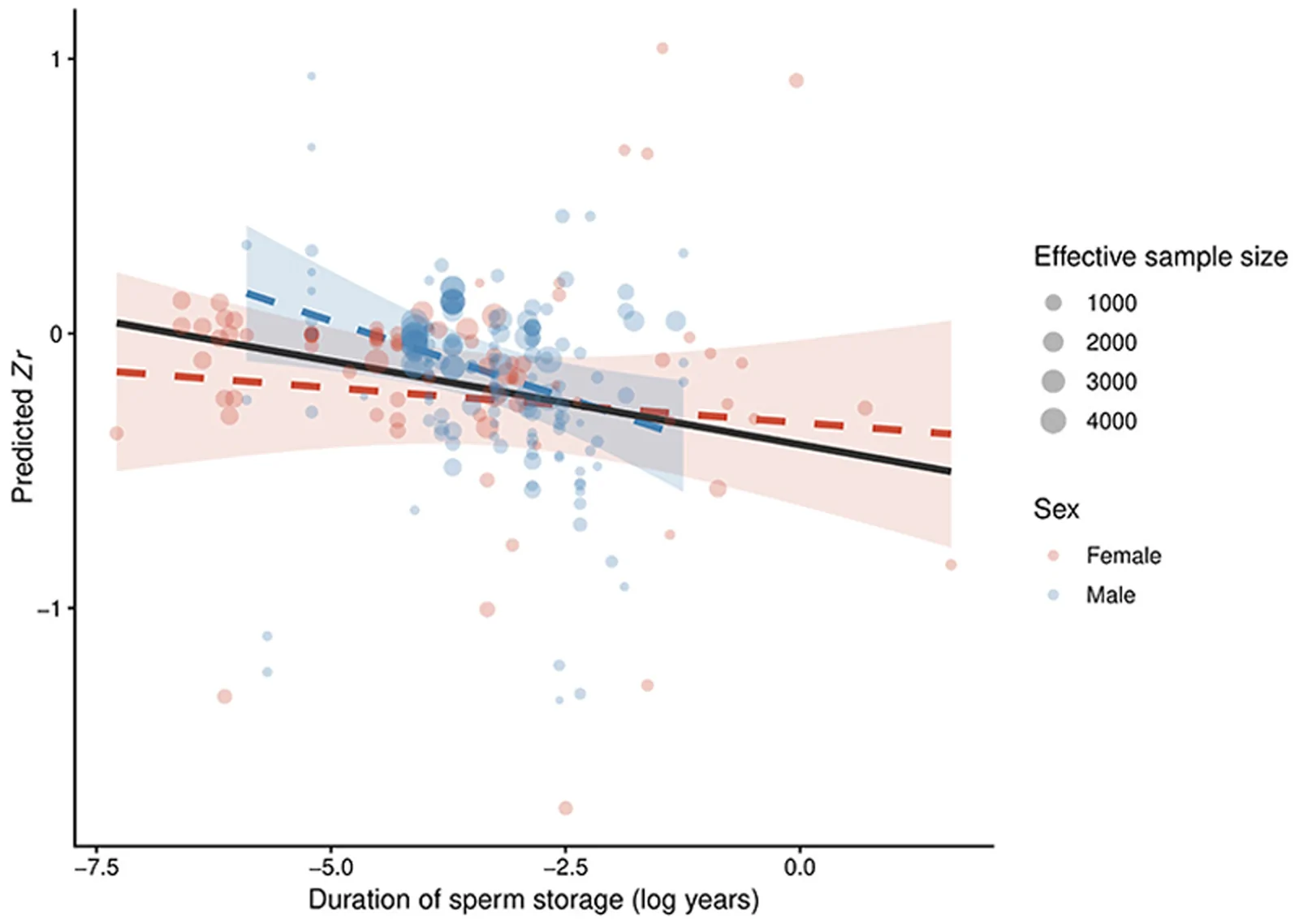
The absolute duration (range) of sperm storage sampled by a study negatively impacts reproductive outcomes (*Zr*) overall, and in male and female animals. TThe size of each data point reflects the effective sample size. 1 day ≈ −5.9 log_e_ years, 1 week ≈ −3.96 log_e_ years, 1 month ≈ −2.485 log_e_ years, 1 year ≈ 0 log_e_ years.

Despite the overall mean effect size across outcomes being more negative for females than males, this difference was not significant (*Q*_*M*_ = 1.355, *Q*_*E*_ = 1347.467, *p* = 0.246, *R*^2^ = 2.365%) and storage in both sexes was deleterious (male: *p* = 0.035; female: *p* < 0.001; figure 3A). The impact of sperm storage tended (non-significantly) to depend on the measured trait (*Q*_*M*_ = 13.976, *Q*_*E*_ = 1280.619, *DF*_8,226_, *R*^2^ = 6.429%; *p* = 0.082, figure 3B, electronic supplementary material, figure S9A). Sperm viability, velocity, motility, morphology, fertilization success and embryo quality were all impacted negatively (figure 3B). Sperm DNA damage, oxidative stress and offspring quality were not significantly influenced (figure 3B). Both the range of absolute (directly from study) and relativized (by species lifespan) durations for which sperm were stored significantly impacted outcomes negatively (*R*^2^_absolute_ = 5.286%; *R*^2^_relative_ = 8.047%; *P*_absolute_ = 0.032; *P*_relative_ = 0.034). Importantly, longer sperm storage was more detrimental in males than it was in females, whereas shorter sperm storage was more detrimental in females than males (figure 4). Population setting did not significantly modulate outcomes despite lab animals experiencing more negative effects than wild, captive or domestic animals (*R*^2^ = 4.729 %, *p* = 0.573, electronic supplementary material, figure S9B). Taxonomic class (*R*^2^ = 0.741%, *p* = 0.996, electronic supplementary material, figure S9C) or sampling method (*R*^2^ = 0.357%, *p* = 0.652, electronic supplementary material, figure S9D) did not explain significant heterogeneity.

### (c) Publication bias

We found no statistical evidence of publication bias in the human (electronic supplementary material, figure S10) or non-human animal datasets (electronic supplementary material, figure S11) when assessed using: visual inspection of funnel plots; aggregated trim-and-fill models (zero estimated missing studies in humans (s.e. = 5.459) and non-human animals (s.e. = 3.994)); small study analysis (humans: *t* = −1.492, *p* = 0.136; non-human animals: *t* = −0.820, *p* = 0.413) and time lag bias analysis (humans: *t* = −0.617, *p* = 0.548; non-human animals: *t* = 0.498, *p* = 0.619). However, the selection model (humans and non-human animals: *p* = 0.001) showed that larger effect sizes have a lower probability of being selected.

## 4 Discussion

Extended *in vivo* sperm storage in males and females occurs frequently across the animal kingdom [1–3]. Despite this, surprisingly little animal-wide quantitative synthesis exists on the mechanisms of sperm damage during storage or how storage impacts sperm performance, including fertilization success or post-fertilization outcomes. Our meta-analysis found significant, but weak (in men) to moderate (in non-human animals) evidence for deleterious effects of *in vivo* sperm storage on various outcomes.

### (a) Mechanisms of trait-specific deterioration

Senescence in somatic cells is caused by advancing organismal age and has several hallmarks: dysregulation of intercellular communication, telomere attrition, mitochondrial dysfunction, epigenetic alterations, increased ROS production, mutation accumulation, reduced proteostasis, chromatin misfolding and mitotic cell cycle arrest [71]. However, only some of these mechanisms explain PMSS, which results from time-dependent deterioration of stored sperm independent of organismal ageing [2,3]. For example, mature sperm are terminally differentiated gametic cells that do not divide further, probably making senescence owingto mitotic arrest less relevant. Sperm also have most of their histones replaced by protamines [24]; thus, the epigenetic cellular mechanism of senescence involving histone modification might be less relevant for sperm. Instead, their lack of cytoplasm (which contains antioxidants and DNA repair machinery), their high metabolic activity and their limited access to energy reserves such as glycogen make DNA damage, oxidative stress and loss of energy reserves the most likely mechanisms of PMSS [2,7,16,18,22,23,25,26,72–83].

In line with predictions from previous studies on the mechanisms of stored-sperm senescence [1,2,22,23,25,26,33,49,81–83], increased sperm DNA damage and oxidative stress were the strongest deleterious effects observed in men (figure 1). We also found evidence that sperm viability and motility declined considerably in men. This outcome might reflect depletion of sperm energy reserves [25] or ROS-mediated damage to the mitochondrial electron transport chain and membrane lipids [26]. Both processes can impair sperm performance and potentially key mechanisms of PMSS. Moreover, as observed in men, sperm motility and viability declined in non-human animals as well, suggesting that these are animal-wide hallmarks of PMSS. Importantly, we also found a decline in fertilization success and embryo quality in non-human animals, highlighting a direct fitness consequence of sperm storage.

The lack of significant effects on some traits might be owing to other factors. First, several traits with non-significant effects had lower sample sizes (e.g. *N*_velocity_ = 13, *N*_Fertilization success_ = 9, *N*_Embryo quality_ = 15, studies). Similarly, among non-human animals, traits represented by few studies, such as DNA quality and oxidative stress, were not significantly impacted (figure 3). Additionally, the lack of effect on sperm morphology, despite the large sample size, might reflect that morphological defects arise during spermatogenesis (e.g. incomplete division, individualization or cytoplasm removal) [84], rather than during storage in men. Trait-specific variation in the effects of storage could also reflect distinct selection pressures on different sperm traits, depending on their relative importance for fertilization success [85]. Additionally, trait-specific effects of sperm storage probably reflect differences in the onset, trajectory and rate of deterioration across traits and outcomes [3,51,52]. Differences between the effects of storage on sperm traits and on fertilization or post-fertilization outcomes might further reflect selection against poor-quality sperm. For instance, if sperm with low velocity are less likely to fertilize eggs [19–21], then even if velocity deteriorates during storage, post-fertilization outcomes such as embryo quality might remain unaffected [52]. Similarly, if sperm-derived DNA damage is repaired by females or embryos [75–80], then offspring quality might not be impacted despite deterioration in stored sperm [50]. Several factors that we did not consider might also explain trait-specific deterioration. For instance, oxidative stress and DNA damage in sperm might be buffered in hibernating species where sperm metabolism is lowered [5,6,86]. Similarly, sperm longevity and activation timing might be important if longer-lived sperm or sperm that remain inactive inside females are less impacted than shorter-lived sperm [1,33].

### (b) Humans versus non-human animals, males versus females

Differences between human and non-human animal studies might also arise from methodological differences. For instance, human studies included individuals who were both infertile and healthy, whereas non-human animal studies did not contain such distinctions. Additionally, human studies focused on male sperm storage only, whereas non-human animal studies included both male and female storage.

In non-human animals, we found sex-specific differences in how storage duration impacted overall outcomes (figure 4). For shorter sperm storage durations, trait deterioration was lower in males; however, under longer sperm storage durations, deterioration was lower in females. A possible explanation is that males have adaptations to maintain sperm quality over the short-but not the long term, while females have adaptations to facilitate long-but not short-term storage [1,13,26,33,49]. Specifically, reproductive fluid and highly specialized sperm storage organs in females might buffer such long-term declines and facilitate sperm preservation over longer periods [1,33,47,48]. This result might also reflect sperm being ‘stressed’ initially owing to a change in their environment when migrating from males to females but then stabilizing over time [49]. It could also reflect sperm being stored in a general organ in females initially (e.g. bursa) but later being moved into more specialized storage organs (e.g. spermathecae) [1,11,13,33,47]. However, these sex-specific results could also reflect differences between male and female data, such as females having greater variance and higher means in their storage duration than males; taxonomic groups and measured outcomes differing between sexes; or that only one study directly compared males and females [81].

**Figure 5:**
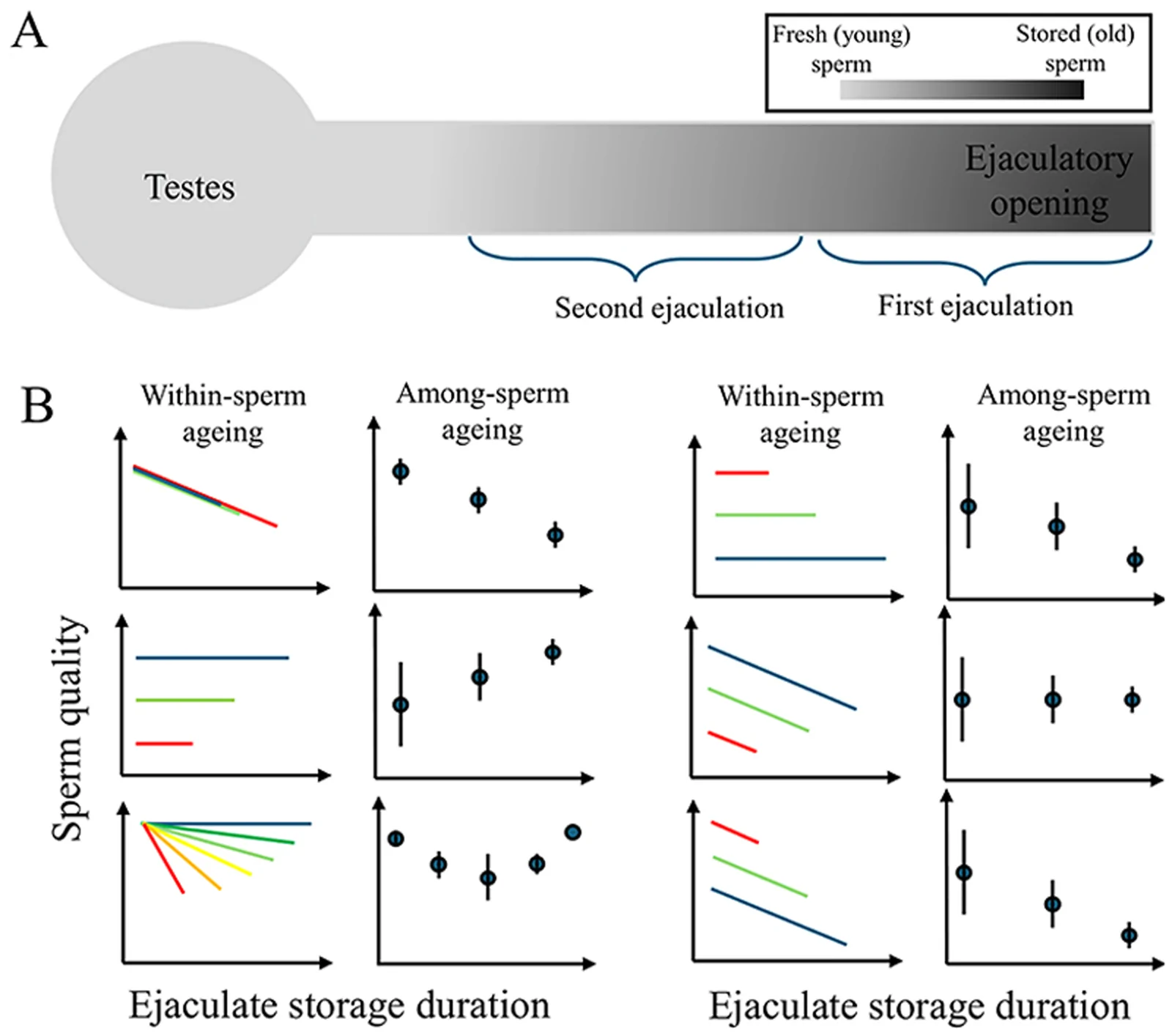
Change in sperm quality during storage depends on sperm stratification, among-sperm variation and within-sperm change in quality. (A) Sperm stratification results in older sperm being closer to the ejaculatory opening. In longitudinal studies, the first ejaculation thus contains older sperm than the second ejaculation. However, the duration of sexual rest when obtaining the ejaculates does not always correspond to the exact age of the sperm unless males have been fully ejaculate-depleted first. By contrast, under sperm mixing, all subsequent ejaculates will have sperm of the same age. (B) Effects of sperm storage depend on among-sperm and within-sperm variation in quality over storage time, akin to selective disappearance and senescence. Each coloured line represents a hypothetical individual sperm, with blue representing longer-lived, and red, shorter-lived, sperm; dots and error bars show ejaculate-level means and variance. Termination of a line represents a sperm’s death/reabsorption.

A key distinction between sperm storage in males and females is that the duration of storage, as measured in studies, reflects post-meiotic sperm age less accurately in males than in females (Box 1). For example, several longitudinal studies in men often collected a first ejaculate after prolonged periods of sexual abstinence and then a second ejaculate after a shorter duration of abstinence [92,93]. Here, the second ejaculate would on average have been stored for a shorter duration and thus would contain more freshly produced sperm than the first. However, because men were unlikely to be completely sperm depleted during their first collection, the measured duration of shorter sexual abstinence in the study would not precisely equate to the true average ‘age’ of sperm in the second ejaculate (Box 1, figure 5A). Even in non-human animal studies that completely depleted males of their stored ejaculates [10,21], ongoing spermatogenesis and sperm reabsorption could alter the average age of sperm in the sampled ejaculates. However, in females, the duration for which sperm were stored, i.e. the timing between mating and fertilization, would perfectly correspond to the minimum post-meiotic age of individual sperm. These differences might further explain the sex-specific effects of storage duration. Future studies could investigate the effects of sperm storage in women by manipulating the timing of sperm inseminated prior to ovulation. Given our results, we also suggest that studies could quantify whether *in vivo* storage of oocytes/eggs, beyond the confounded influence of maternal age [89] might impact reproductive outcomes [17].

### (c) Evolutionary consequences

The ability to store sperm for extensive durations provides an evolutionary advantage by allowing the temporal separation of spermatogenesis, ejaculation and fertilization [12]. However, sperm also deteriorate during storage owing to PMSS, as demonstrated in our study. Thus, animals have evolved several strategies to alleviate these effects. For example, unlike DNA in other cells, sperm have tightly bound chromatin that reduces the amount of DNA exposed to ROS [72,74,75,78]. Additionally, in some species, both males and females spontaneously discard old sperm [35–38]. An extreme example of this is male masturbation, hypothesized to have evolved as an adaptation for ejecting stored, old sperm [36], which we predict is more likely to occur in species where sperm are stratified by age [3] (figure 5A, Box 1). Additionally, secretion of antioxidants and small RNA, as well as the evolution of specialized sites or organs for sperm storage in both males and females (see electronic supplementary material, Supplementary text S10), suggest strong selection pressures to preserve stored sperm and alleviate PMSS [1,13,33,43–48,73–77].

We predict that PMSS is likely to have important consequences for sexual selection—specifically, polyandry, cryptic female choice and last-male sperm precedence. For example, females might be temporally partitioning sperm from different males in their storage organs [92,94,95] to preferentially use fresh sperm. In taxa where sperm are stratified in males and the first ejaculate contains older sperm than subsequent ejaculates, females might also benefit from re-mating with the same male.

Females could also be biassing paternity towards last-mated males owing to sperm from first-mated males having been stored for extended durations [43,95]. Our meta-analysis shows that variation occurs not only between ejaculates but also over time within an ejaculate, and that both forms of variation are important for sexual selection. Crucially, we propose that mortality of poor-quality sperm during storage might generate differences between within- and among-individual patterns of post-meiotic sperm ageing, analogous to selective disappearance (Box 1, figure 5B), which has major implications for sperm selection and reproductive health [88–91].

### (d) Biomedical consequences

Our results qualitatively agree with other meta-analyses that synthesized a smaller sample size of studies and used simpler models, but that also found deleterious effects of sperm storage in men [16,22,23,96]. Therefore, these results collectively have important implications for human and non-human animal fertility and captive breeding programmes. We recommend that sperm storage timeframes should depend on whether the goal is to optimize sperm quantity, sperm quality or both. This follows from the fact that sperm storage induces PMSS, but sexual rest in many male animals, including men, also increases sperm numbers [10,16,22,52,97,98]. Therefore, in scenarios where whole ejaculates are used for fertilization, such as IVF [99], optimizing both sperm number and quality will probably require intermediate durations of sperm storage or sexual abstinence. On the other hand, when a single high-quality sperm is required for fertilization, as in intracytoplasmic sperm injection [100], shorter durations of sperm storage, as opposed to the 2–7 days recommendation by the W.H.O [101], might be preferable. However, because the overall effect size in men was weak despite being significant, our recommendations should be applied with caution.

## Supplementary Material

Electronic supplementary material is available online at https://doi.org/10.6084/m9.figshare.c.8337057.

Supplementary figures and tables

## Data Availability

All data associated with our study, pre-registration of study and the code used to analyse these data are available at Open science framework with the link https://osf.io/jxv7z/files/osfstorage and via Dryad [102]. Supplementary material is available online [103].

## Box 1 Storage might not necessarily cause PMSS.

Despite individual sperm ageing during storage, the average age of ejaculates might not increase in males, and sperm might not deteriorate for several reasons. First, if sperm are stratified such that freshly produced sperm are located distally (near the testes) and older sperm are located caudally (closer to the site of ejaculation) [3,87], then increasing duration of sexual abstinence would lead to ejaculated sperm being older than unejaculated sperm or than sperm collected in successive ejaculations (figure 5A). However, if freshly produced sperm mix with old sperm, this could prevent the average age of the collected ejaculate from increasing over sexual abstinence, and sperm in successive ejaculates would be of the same average age [3].. Second, selective disappearance might buffer senescence of stored sperm. Specifically, if among-sperm variation generates non-random sperm death, then surviving sperm sampled at older post-meiotic ages would represent a biased cohort of high-quality sperm [88–91] (figure 5B).
